# Itaconate inhibits ferroptosis of macrophage via Nrf2 pathways against sepsis-induced acute lung injury

**DOI:** 10.1038/s41420-021-00807-3

**Published:** 2022-02-02

**Authors:** Ruyuan He, Bohao Liu, Rui Xiong, Boxin Geng, Heng Meng, Weichen Lin, Bo Hao, Lin Zhang, Wei Wang, Wenyang Jiang, Ning Li, Qing Geng

**Affiliations:** 1 Department of Thoracic Surgery, Renmin Hospital of Wuhan University, Wuhan, China; 2 School of Basic Medicine, Army Medical University (Third Military Medical University), Chongqing, China

**Keywords:** Cell death, Respiratory tract diseases

## Abstract

Itaconate, a metabolite produced during inflammatory macrophage activation, has been extensively described to be involved in immunoregulation, oxidative stress, and lipid peroxidation. As a form of iron and lipid hydroperoxide-dependent regulated cell death, ferroptosis plays a critical role in sepsis-induced acute lung injury (ALI). However, the relationship between itaconate and ferroptosis remains unclear. This study aims to explore the regulatory role of itaconate on ferroptosis in sepsis-induced ALI. In in vivo experiments, mice were injected with LPS (10 mg/kg) for 12 h to generate experimental sepsis models. Differential gene expression analysis indicated that genes associated with ferroptosis existed significant differences after itaconate pretreatment. 4-octyl itaconate (4-OI), a cell-permeable derivative of endogenous itaconate, can significantly alleviate lung injury, increase LPS-induced levels of glutathione peroxidase 4 (GPX4) and reduce prostaglandin-endoperoxide synthase 2 (PTGS2), malonaldehyde (MDA), and lipid ROS. In vitro experiments showed that both 4-OI and ferrostatin-1 inhibited LPS-induced lipid peroxidation and injury of THP-1 macrophage. Mechanistically, we identified that 4-OI inhibited the GPX4-dependent lipid peroxidation through increased accumulation and activation of Nrf2. The silence of Nrf2 abolished the inhibition of ferroptosis from 4-OI in THP-1 cells. Additionally, the protection of 4-OI for ALI was abolished in Nrf2-knockout mice. We concluded that ferroptosis was one of the critical mechanisms contributing to sepsis-induced ALI. Itaconate is promising as a therapeutic candidate against ALI through inhibiting ferroptosis.

## Introduction

As a life‐threatening condition, sepsis is the foremost contributor to hospital death. Sepsis-induced injury, shock, and dysfunction of multiple organs remain the major cause of death in septic patients [1]. The lungs are particularly susceptible to injury during sepsis, and the primary risk factors of acute lung injury (ALI) in >50% patients were attributed to sepsis [2]. However, the pathophysiology and pathogenesis of sepsis-induced ALI are not fully understood. As the most common lung immune cells at homeostasis [3], macrophage plays a crucial role in sepsis-induced ALI. The recruited and activated macrophages by lipopolysaccharide (LPS) and originally resident alveolar macrophages can release pro-inflammatory cytokines and induce neutrophil infiltration [4], further aggravating inflammation, destruction of the endothelial barrier, and blockage of pulmonary microcirculation, intensifying lung injury [5].

Ferroptosis, a novel form of iron and lipid hydroperoxide-dependent regulated cell death, is distinct from apoptosis, autophagy, or other forms of cell death [6]. It has been implicated in pathological cell death associated with all kinds of diseases, including degenerative diseases, carcinogenesis, and ischemia-reperfusion injury. Recently, increasing evidence indicated that ferroptosis plays an important role in sepsis-induced multiple organ injury and the inhibition of ferroptosis can significantly alleviate organ injury, including cardiac injury [7], acute lung injury [8, 9], liver injury, and acute kidney injury [10]. Ferrostatin-1, the inhibitor of ferroptosis, was found to rescue the downregulation of ferroptosis markers including cystine/glutamate transporter (SLC7A11) and glutathione peroxidase 4 (GPX4) in LPS-induced ALI [8]. Beyond that, ferroptosis was also found to contribute to ischemia reperfusion-induced ALI, radiation-induced lung injury, and seawater drowning-induced acute lung injury [11–13]. The inhibition of ferroptosis may serve as a novel potential therapeutic strategy for ALI.

Itaconate, a metabolite synthesized by the enzyme encoded by IRG1 [14], is produced by diverting aconitate away from the tricarboxylic acid cycle (TAC) during macrophage activation [15]. Ferroptosis has been extensively described to be involved in cell metabolism, oxidative and electrophilic stress responses, and immune responses [15]. Nuclear factor erythroid 2-related factor 2 (NFE2L2, or Nrf2), a transcription factor, is a key regulator of the cellular antioxidant response and played a critical role in regulating lipid peroxidation and iron metabolism through affecting the transcription of ferroptosis-associated genes [16]. Recently, many studies have clarified that itaconate can attenuate reactive oxygen species (ROS) production and lipid peroxidation [17, 18]. 4-octyl itaconate (4-OI), a cell-permeable derivative of endogenous itaconate, has the potential to activate Nrf2 pathways [19]. However, the possible capacity of itaconate in inhibiting ferroptosis has not been explored yet. In this study, we demonstrated that itaconate can alleviate sepsis-induced ALI by inhibiting ferroptosis of macrophages in a Nrf2-dependent manner.

## Results

1. Itaconate alleviates sepsis-induced ALI and macrophages infiltration in lung.
  First, we investigated the function of 4-OI in ALI model mice. Compared to LPS group, pre-treatment of 4-OI significantly attenuated LPS-induced ALI, as reflected by pulmonary hemorrhage, interstitial edema, thickening of the alveolar wall, and tissue damage. Masson staining showed that 4-OI also reduced the level of LPS-induced lung interstitial fibrosis (Fig. 1A). Besides, the lung injury score of 4-OI pre-treatment group is significantly lower than LPS group (Fig. 1B). The increase of lung wet/dry weight ratio was reversed by the application of 4-OI (Fig. 1C). Similarly, 4-OI significantly prevented LPS-induced inflammatory response in lung tissues, reduced protein and mRNA levels of TNF-α, IL-1β and IL-6 (Fig. 1D–G). The immunostaining of macrophage cell marker CD68 was used to identify macrophages and the result demonstrated that 4-OI can significantly reduce LPS-induced macrophage infiltration in lung tissue (Fig. 1H). Taken together, these data unveiled that itaconate can significantly alleviate the ALI and macrophage infiltration induced by sepsis in mice.

**Fig. 1:**
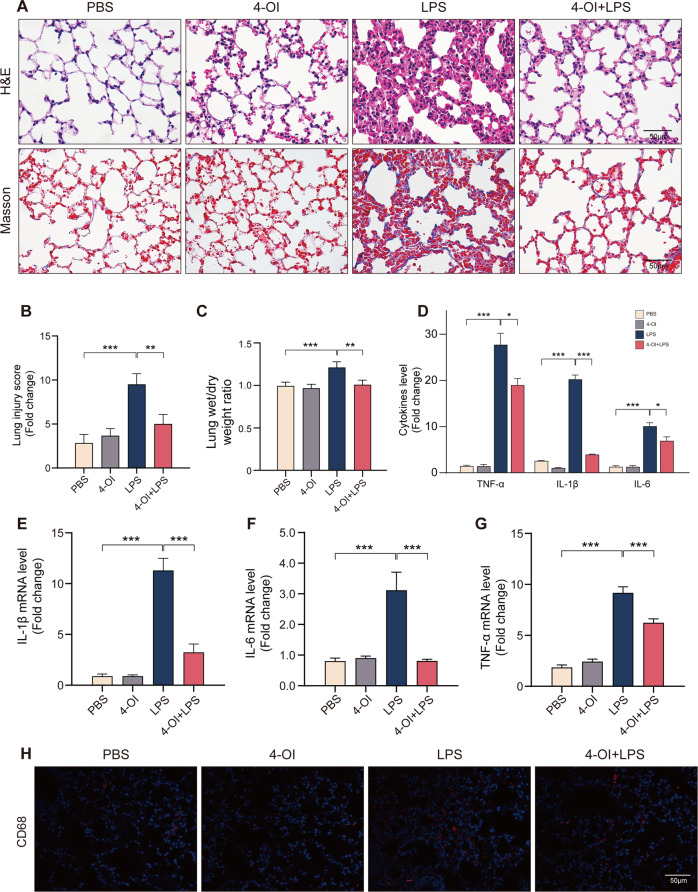
4-OI significantly alleviates sepsis-induced ALI in vivo. **A** Representative images of H&E and Masson staining of lung tissue. Morphology was examined using light microscopy. **B** Semiquantitative histological scores of lung injury in groups described in panel (*n* = 6). **C** Lung wet-to-dry weight ratio and was determined in all groups (*n* = 6). **D** ELISA for TNF-α, IL-1β and IL-6 in murine lung tissue (*n* = 6). **E**–**G** Relative levels of TNF-α, IL-1β and IL-6 mRNAs in murine lung tissue (*n* = 6). **H** Representative images of immunofluorescence staining for CD68 and DAPI in lung tissue. (Data are presented as Mean ± SD. *p < 0.05, **p < 0.01, ***p < 0.001).
2. Itaconate inhibits ferroptosis in mice with sepsis-induced ALI
  To address the role of itaconate in ferroptosis of ALI. We performed differential gene expression analysis using macrophage RNA-seq data from previous studies [20]. We found that ferroptosis-associated genes existed significantly differences after itaconate derivatives pretreatment prior to LPS stimulation (Fig. 2A). Consistently, some ferroptosis-associated gene also significantly changed in IRG1-KO macrophage compare [21] (Fig. S1A). It is well known that the glutathione (GSH)-dependent antioxidant enzyme GPX4 is presumed to play a central role in blocking ferroptosis. We detected the expression of GPX4 and PTGS2 level, 2 well-accepted markers of ferroptosis, in lung tissues, and found that the protein and mRNA expressions of GPX4 decreased and PTGS2 increased in lung tissues after LPS administration (Fig. 2B–D). Meanwhile. 4-OI pretreatment can significantly increase GPX4 and decrease PTGS2 (Fig. 2B–D). Besides, 4-OI can reduce LPS-induced increase of level of tissue iron (Fig. 2E). The 4-hydroxy-2-nonenal (4-HNE) and malondialdehyde (MDA), which were principal aldehydic metabolites from lipid peroxidation process, were also assessed. The MDA level (Fig. 2F) and 4-HNE (Fig. 2G) staining confirmed that LPS can promote lipid peroxidation in lung, but 4-OI can significantly inhibit it. In brief, itaconate significantly inhibited the ferroptosis of ALI induced by sepsis in mice.

**Fig. 2:**
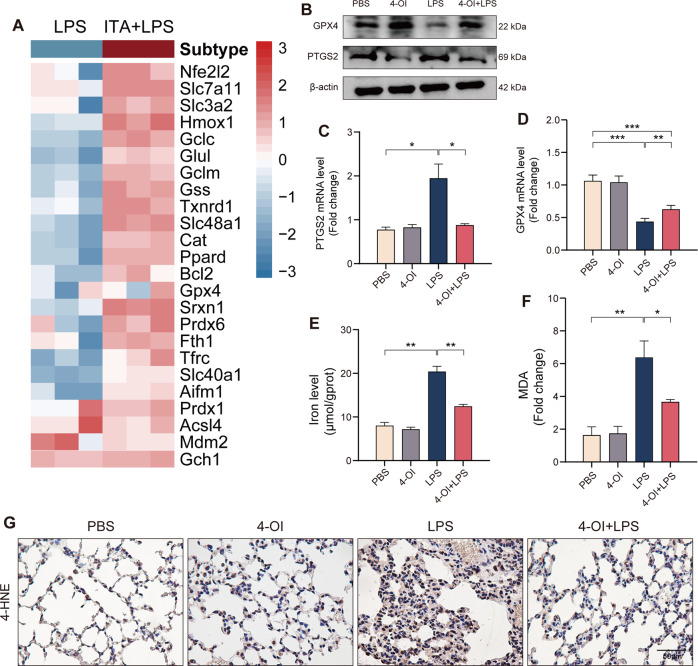
4-OI inhibits ferroptosis in murine lung during sepsis-induced ALI. **A** Heatmap of selected ferroptosis associated genes in macrophage between LPS and Itaconate + LPS (4 h) group. **B** Western blots for GPX4 and PTGS2 in murine lung tissue. **C**, **D** Relative mRNA levels of GPX4 and PTGS2 in murine lung tissue (*n* = 6). **E**, **F** Relative iron and MDA levels of murine lung tissue (*n* = 6). **G** Representative images of immunohistochemistry staining for 4-HNE in murine lung tissue. (Data are presented as Mean ± SD *p < 0.05, **p < 0.01, ***p < 0.001).
3. Itaconate inhibit ferroptosis through increasing and activating Nrf2 in sepsis-induced ALI
  A previous study has indicated that 4-OI can directly alkylate multiple cysteine residues of protein KEAP1, reducing Nrf2 degradation and increasing the expression of downstream genes [19]. We found that 4-OI boosted Nrf2 protein level (Fig. 3A), without affecting its mRNA level (Fig. 3B); 4-OI also reversed LPS-induced Nrf2 reduction and increased the expression of recognized downstream target genes HO-1 (Fig. 3A, C). Next, we furthered investigated the regulation of Nrf2 on lipid peroxidation, a crucial process in ferroptosis. To eliminate phospholipid peroxides against ferroptosis, GPX4 uses the glutathione generated from cysteine with the assistance of enzyme glutamate–cysteine ligase (GCL). The cysteine mainly obtained from extracellular cystine through system X_C_^−^, which exchanges intracellular glutamate for extracellular cystine. Our results showed that 4-OI can significantly elevate SLC7A11 and GCLM levels and reverse their decrease induced by LPS (Fig. 3A, D, E), which are subunits of GCL and X_C_^−^ and can be controlled by Nrf2 as previous studies, respectively. Consistently, 4-OI pretreatment also increases the GSH level in vivo after LPS stimulation and GSH/GSSG ratio (Fig. 3F, G). The immunofluorescence staining indicated that the LPS induced ROS increase was significantly inhibited by 4-OI (Fig. 3H, I). In conclusion, itaconate inhibited the ferroptosis through increasing and activating the Nrf2 in mice.

**Fig. 3:**
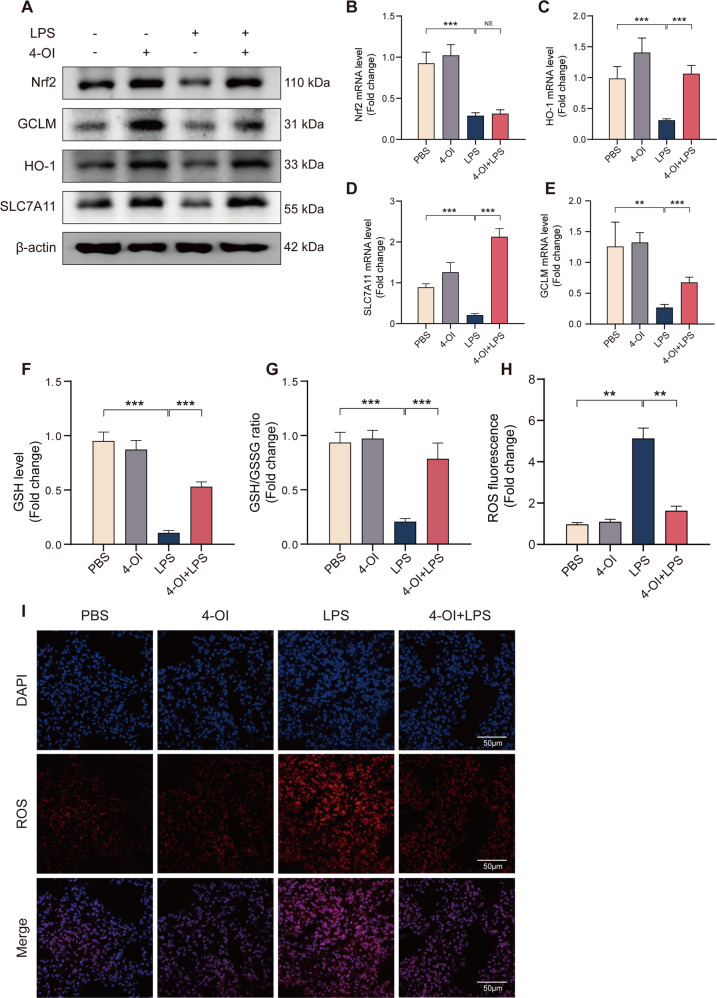
4-OI inhibits ferroptosis through increasing and activating Nrf2 in vivo during ALI. **A** Western blots for Nrf2, GCLM, HO-1 and SLC7A11 in murine lung tissue. **B**–**E** Relative mRNA levels of Nrf2, GCLM, HO-1 and SLC7A11 in murine lung tissue (*n* = 6). **F**, **G** Relative levels of GSH and GSH/GSSG ratio in murine lung tissue (*n* = 6). **H**, **I** Representative images of fluorescence probe for ROS and its statistical results (*n* = 6) in lung tissue. (Data are presented as Mean ± SD *p < 0.05, **p < 0.01, ***p < 0.001).
4. Itaconate increases the level of Nrf2 to inhibit LPS-induced ferroptosis of THP-1 cell
  Macrophages play a crucial role in ALI [3]. Consistent with other studies, LPS significantly increases macrophage infiltration in lung tissue (Fig. 1H). Subsequently, we conducted in vitro experiments on THP-1 cells to assess the relationship between LPS-induced ALI and ferroptosis. GPX4 protein levels were determined at 0 h, 1 h, 3 h, 6 h, 12 h and 24 h after LPS stimulation, and increased sharply in 1 h then persistently reduced until 24 h (Fig S1B). To further evaluate the function of 4-OI inhibited LPS-induced ferroptosis. Ferrostatin-1 (Fer-1), a typical ferroptosis inhibitor that can block lipid peroxidation, was used to treat THP-1 before LPS stimulation. Different concentrations of 4-OI pretreatment were also used referring to a previous study [21]. Our results indicated that both 4-OI and Fer-1 pretreatment can significantly increase the expression of Nrf2, GPX4, SLC7A11C and GCLM, and 0.25 mM exhibited more obviously ferroptosis resistance (Fig. 4A). Compared to LPS group, The GSH and GSH/GSSG ratio were elevated in 4-OI or Fer-1 pretreatment group (Fig. 4B, C). The 4-OI also significantly reduced the cell death, MDA and ROS level induced by LPS (Fig. 4D–G). To further confirm that the ferroptosis resistance of 4-OI was dependent on Nrf2, the siRNA against Nrf2 was constructed and successfully reduced Nrf2 levels in THP-1 cells (Fig. 4H). After the silence of Nrf2, the GPX4 level significantly reduced and the protection of 4-OI was almost completely abolished (Fig. 4I). Consistently, the protein and mRNA levels of GCLM, SLC7A11 and HO-1 decreased and can’t be reserved by 4-OI (Fig. 4J–M). In summary, we confirmed that the ferroptosis resistance of itaconate was dependent on Nrf2 and almost completely abolished after silence of Nrf2.

**Fig. 4:**
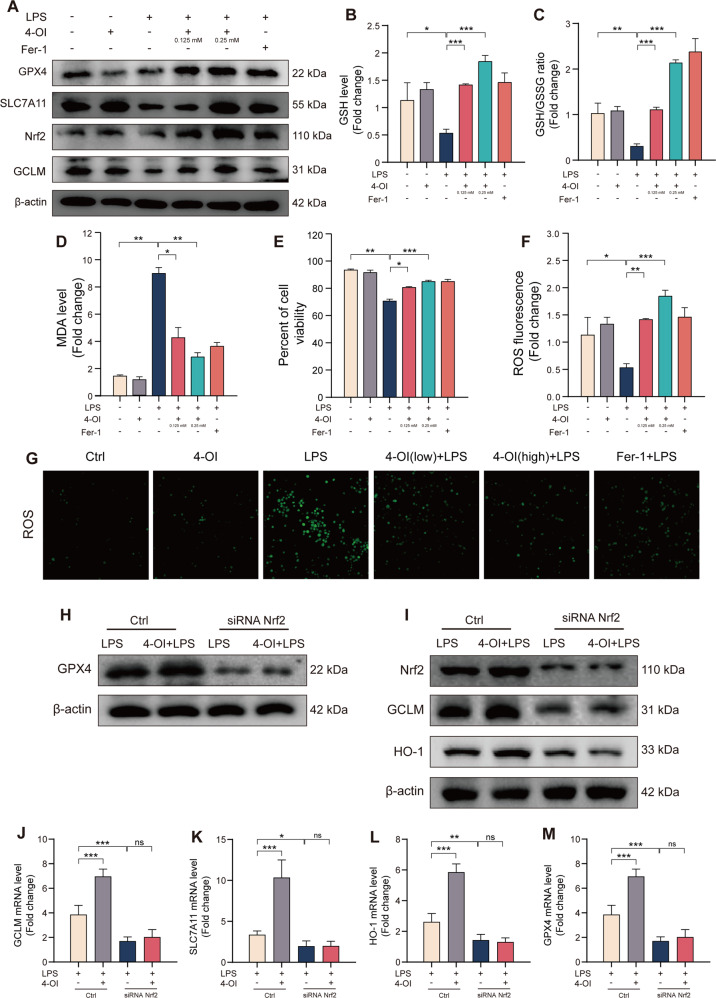
4-OI inhibits LPS induced ferroptosis through Nrf2-dependent pathways in THP-1 cell. **A** After induced into macrophage-like state through 100 ng/ml PMA (6 h), THP-1 cells were harvested following 4-OI pretreatment (12 h) and LPS (3 h), immunoblotted for the GPX4, Nrf2, GCLM and SLC7A11 level. **B**, **C** Relative level of GSH and GSH/GSSG ratio in THP-1 cell lysates. **D** Relative MDA level in THP-1 cell lysates. **E** After 4-OI pretreatment (12 h) and LPS (3 h), Trypan Blue staining was used to determine THP-1 cell viability (*n* = 6). **F**, **G** Representative images of fluorescence probe for ROS and its quantitative results in THP-1 cell (*n* = 6). **H**, **I** Western blots for GPX4, Nrf2, GCLM and HO-1 in THP-1 cells transfected with siRNA against Nrf2. J-M Relative mRNA levels of GPX4, Nrf2, GCLM and HO-1 in THP-1 cells with or without si-Nrf2 transfection (*n* = 6). (Data are presented as Mean ± SD *p < 0.05, **p < 0.01, ***p < 0.001).
5. The protection of 4-OI against ferroptosis in ALI was abolished in Nrf2-KO mice.

**Fig. 5:**
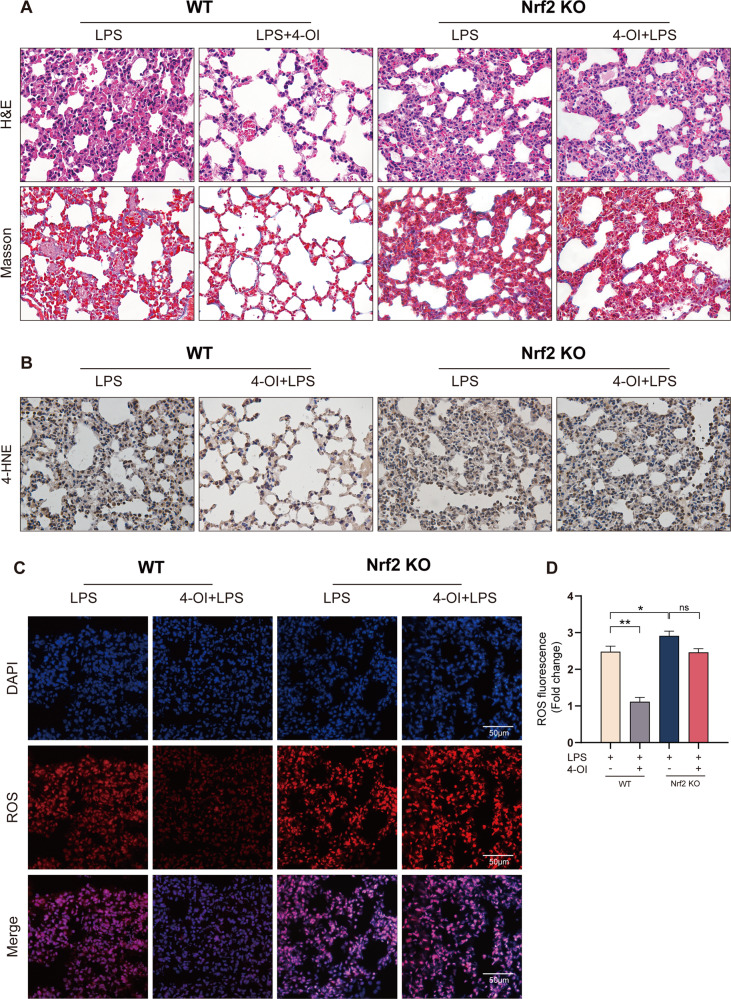
The protection of 4-OI for sepsis-induced ALI was abolished in Nrf2-KO mice. **A** Representative images of H&E and Masson staining of murine lung tissue. **B** Representative images of immunohistochemistry staining for 4-HNE in murine lung tissue. **C**, **D** Representative images of fluorescence probe for ROS and its quantitative results in murine lung tissue (*n* = 6). (Data are presented as Mean ± SD *p < 0.05, **p < 0.01, ***p < 0.001).

**Fig. 6:**
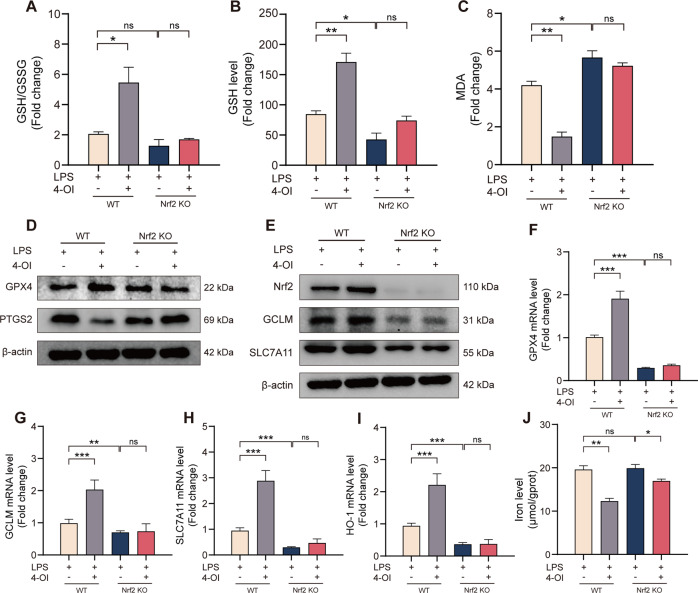
The inhibition of 4-OI for ferroptosis was abolished in Nrf2-KO mice. **A**–**C** Relative GSH, GSH/GSSG ratio and MDA levels in murine lung tissue (*n* = 6). **D**, **E** Western blots for GPX4, PTGS2, Nrf2, GCLM and SLC7A11 in murine lung tissue. **F**–**I** Relative mRNA levels of GPX4, GCLM, SLC7A11 and HO-1 in murine lung tissue (*n* = 6). **J** Relative iron levels of murine lung tissue (*n* = 6). (Data are presented as Mean ± SD *p < 0.05, **p < 0.01, ***p < 0.001).

To further validate that the inhibitions of 4-OI on ferroptosis was in a Nrf2-dependent manner, Nrf2-knockout mice were used to construct ALI models. We found that the deletion of Nrf2 aggravated ALI induced by sepsis (Fig. 5A). However, the protection of 4-OI for ALI was abolished in Nrf2^−/−^ mice. Compared to WT group, the 4-HNE (Fig. 5B) and ROS level was also non-significantly attenuated in the lung tissue (Fig. 5C, D). We also detected the GSH, GSH/GSSG ration, and MDA levels (Fig. 6A–C), deletion of Nrf2 invalidated the anti-lipid peroxidation function of 4-OI. Consistently, the inhibition of 4-OI for ferroptosis was also abolished (Fig. 6D–I). Interesting, 4-OI can still reduce the level of tissue iron induced by LPS in Nrf2^−/−^ mice (Fig. 6J), indicating that 4-OI may also regulate cytoplasmic iron through an Nrf2-independent mechanism. Collectively, we determined that itaconate inhibited ferroptosis of macrophages through blocking Nrf2 degradation and protected against sepsis-induced ALI.

## Discussion

**Fig. 7:**
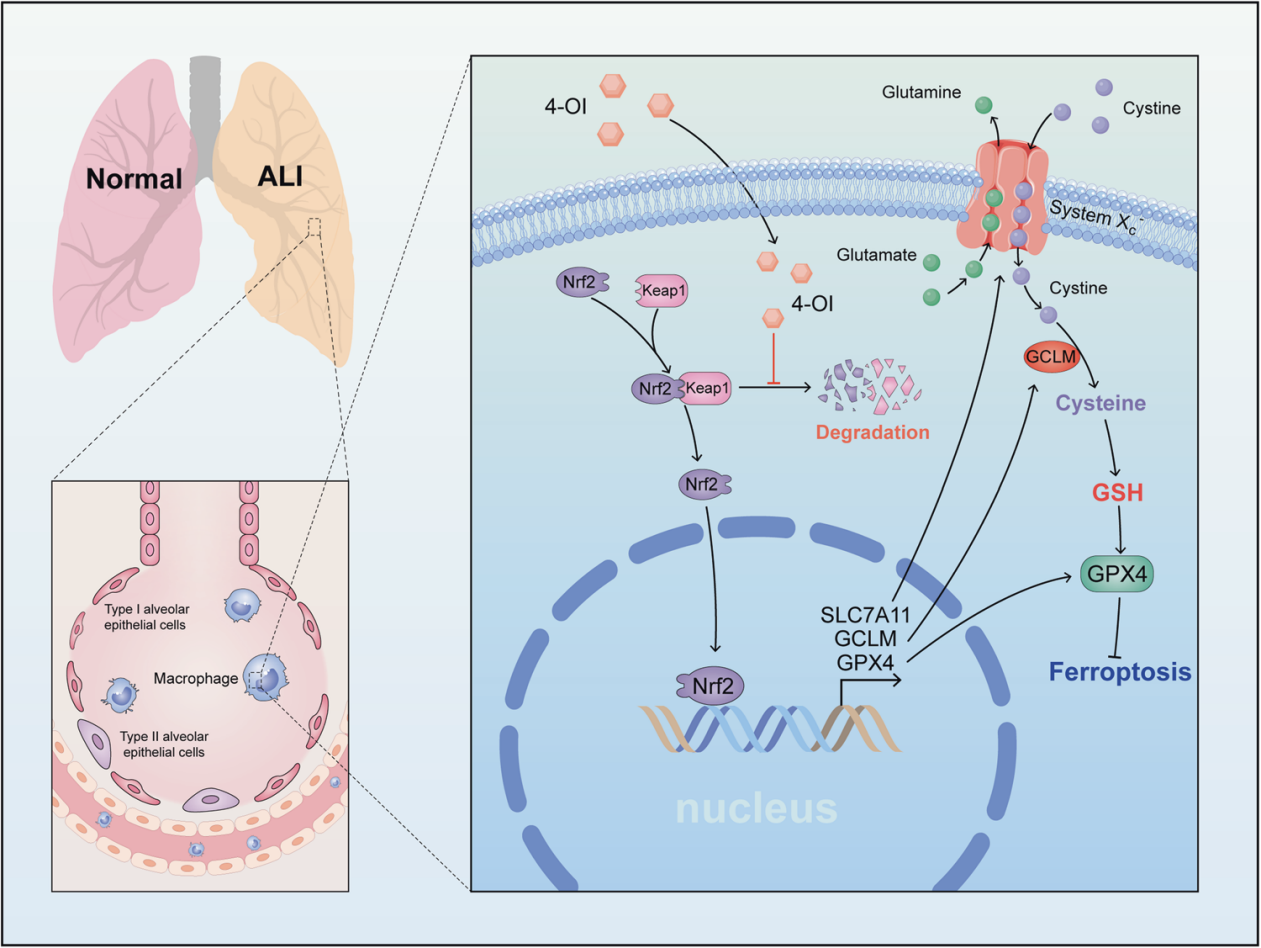
Graphical abstract of 4-OI alleviate sepsis-induced ALI. In macrophages, 4-OI inhibited Nrf2 degradation and promoted the transcription of target genes, including SLC7A11, GCLM and GPX4. They contributed to inhibiting the ferroptosis induced by LPS and alleviated sepsis-induced ALI.

Itaconate is the prime example of metabolic reprogramming in macrophage and is synthesized as a by-product of the Krebs cycle [22]. It has been extensively reported that itaconate can reprogram cell metabolism, regulate inflammation and immune states [15]. In the present study, we demonstrated for the first time that itaconate can alleviate the sepsis-induced ALI through inhibiting ferroptosis of macrophage (Fig. 7). Mechanistically, we identified that the derivative of endogenous itaconate 4-OI can inhibit the GPX4-dependent lipid peroxidation. Moreover, we discovered that the function of 4-OI to inhibit macrophage ferroptosis was dependent on the blockage of the degradation of Nrf2, the resultant increase of Nrf2 promoted the transcription of target genes, including GPX4, GCLM, SLC7A11. Consistent with other studies, we also demonstrated that pro-inflammatory factors, including IL-1β, IL-6, and TNF-α, in the lung is reduced following 4-OI pretreatment in LPS-induced ALI [17–19]. Based on these findings, we supposed that itaconate was a possible inhibitor of ferroptosis against LPS-induced ALI.

Nrf2 is considered a pivotal regulator of the antioxidant defense, as many of its downstream target genes are critical in maintaining redox homeostasis [16]. Due to its multifaceted role in regulating downstream genes, Nrf2 is associated with multiple cell death ways [23]. As a form of regulated cell death driven by iron-dependent lipid peroxidation, regulation of ferroptosis mainly involves three aspects: the abundance of key phospholipid substrates, the factors that drive their peroxidation, such as iron-dependent enzymes and labile iron, and the factors that eliminate these lipid peroxides [24]. Interestingly, multiple genes associated with iron metabolism and lipid peroxidation were transcriptionally regulated by Nrf2. Besides GPX4 and SLC7A11 in the present study, ferroportin (SLC40A1), a protein responsible for the outflux of iron from cell, as well as a heavy chain of ferritin (FTH1), the key cytoplasmic iron storage protein, is also controlled by Nrf2. Thus, targeting the upstream regulators of the ferroptosis through pharmacological modulation of the Nrf2 pathway was one of the optimal approaches for treating ferroptosis-related diseases. Previous research reported that endogenous itaconate and its cell-permeable derivative 4-octyl itaconate (4-OI) can alkylate cysteine residues 151, 257, 288, 273, and 297 on the kelch-like ECH-associated protein 1 (KEAP1) [19], a cytoplasmic protein that can form complexes with Nrf2 and degrade Nrf2 by ubiquitination. Consistently, our study showed that itaconate increased the protein level of Nrf2 without an effect on its mRNA level, which implicated that the reduction of Nrf2 degradation was due to the alkylation of Keap1. Apart from inhibiting LPS-induced ferroptosis, the increase of Nrf2 can also reduce IL-1β expression dependent on hypoxia-inducible factor 1α (HIF1α). Chromatin immunoprecipitation (ChIP)-sequencing experiments performed in BMDMs have also demonstrated that Nrf2 might act as a direct transcriptional repressor of IL-1β [25]. Additionally, we found that 4-OI can still reduce the level of iron LPS in Nrf2^−/−^ mice, indicating that itaconate may regulate the metabolism of iron through other Nrf2-independent pathways. The relationship between 4-OI and iron metabolism needs to be further explored in future studies.

Itaconate was the most abundant metabolite in LPS-treated human macrophages [19]. In addition to alkylating cysteines in this study, itaconate can modify macrophage immune responses and has the potential to significantly influence inflammatory outcomes. Itaconate can inhibit succinate dehydrogenase (SDH), which was an important enzyme in the TCA cycle, and converts succinate to fumarate. SDH can oxidize succinate and produce reduced coenzyme Q, which transfers electrons to complex I and generates ROS to regulate inflammatory response [26]. Besides, itaconate can regulate the inflammatory response by inhibiting glycolysis via targeting GAPDH. Itaconate has also been shown to repress STING and the expression of type I interferons dependent on Nrf2 in response to STING activators and viruses such as herpes simplex virus 1 [27]. In the present study, we first demonstrated that itaconate can inhibit ferroptosis against LPS-induced ALI in an Nrf2-dependent manner. Of note, there are some limitations in this study. whether there is any difference between exogenous itaconate derivatives and endogenous itaconate, such as regulatory mechanisms and targets. We mainly proved that the 4-OI inhibits ferroptosis through repressing the lipid peroxidation, the relationship between itaconate and phospholipid substrates or iron metabolism still needs to be further explored in the future.

## Materials and methods

### Reagents and antibodies

Lipopolysaccharides (LPS) from Escherichia coli O111:B4 (#L2630) was purchased from Sigma-Aldrich (St Louis, MO, USA). 4-Octyl Itaconate (4-OI) was purchased from MCE (Shanghai, China). Primary antibodies against Nrf2 (AF0639) and GCLM (DF7268) were purchased from Affinity Biosciences and GPX4 (14432-1-AP), SLC7A11 (26864-1-AP), HO-1 (10701-1-AP), PTGS2 (12375-1-AP), and CD68 (28058-1-AP), were purchased from Proteintech Group (Wuhan, China). 4-HNE (bs-6313R) for immunohistochemical staining was purchased from Biosss (Beijing, China). Malondialdehyde (MDA) assay kit (A003-1-2), Total glutathione/Oxidized glutathione assay kit (A061-1-1), and tissue iron assay kit (A039-2-1) were purchased from Jiancheng Bioengineering Institute (Nanjing, China). TNF-α, IL-1β, IL-6 ELISA kits were obtained from Cloud-Clone (Wuhan, China). The ROS Fluorescent Probe Kit was used to detect ROS of tissue (KeyGEN, China) and cell (Biosharp, China). Trypan Blue Staining solution (0.4%) was purchased from Biosharp (Hefei, China)

### Animal model of sepsis-induced acute lung injury

All animal procedures were conformed to the Guide for the Care and Use of Laboratory Animals. The present study was approved by the Animal Use Committees of Renmin Hospital of Wuhan University. Male wild-type C57BL/6 mice (6–8 weeks old) were obtained from Hubei Province Experimental Animal Center (Wuhan, China). Nrf2‐KO (Stock no.017009) mice were obtained from the Jackson Laboratory. Animals were maintained under specific pathogen-free conditions. All mice were randomized to each group. Unless otherwise specified, the number of mice in each group is 6. The sepsis-induced acute lung injury (ALI) model was established by intraperitoneal LPS instillation at a dose of 10 mg/kg. For the treatment group, mice were pretreated with 4-OI (25 mg/kg) in 40% cyclodextrin for 2 h before stimulation with LPS intraperitoneally for 2 h as described [19]. If improper intraperitoneal injection operation causes the death of the mouse, exclude and supplement the same amount. After stimulation with LPS for 12 h, the left lung of mice was eviscerated. Formalin was instilled in the trachea to expand the alveoli as described [28].

### Cell culture and transfection

The human monocytic leukemia cell line THP-1 were obtained from the American Type Culture Collection (ATCC, Manassas VA, USA) and was cultured in RPMI 1640 culture medium (Servicebio, China) supplemented with 10% fetal bovine serum (FBS; Gibco, US) and 1% penicillin/streptomycin (P/S) (Biosharp, China), and maintained at 37 °C, 5% CO_2_ humidified incubator. For the siRNA transfections, the cells were transfected with Nrf2 siRNA (5′-AUUGAUGUUUCUGAUCUAUCACUTT-3′). Lipofectamine 3000 (Invitrogen, USA) was used for plasmid transfections according to the manufacturer’s instructions.

### Macrophage differentiation and stimulation

The macrophage-like state was obtained by treating THP-1 monocytes for 6 h with 100 ng/ml 1 phorbol 12-myristate 13-acetate (PMA; MCE) in 6-wells cell culture plates (Corning, US) with 1.5 ml cell suspension in each well. After PMA induced, differentiated macrophage-like THP-1 cells were washed twice with sterile phosphate-buffered saline (PBS; Servicebio, China) and grown in RPMI 1640 medium without PMA but containing 1% P/S and 10% FBS for 24 h. Unless stated, the 1 mg/ml LPS was used to stimulate for 3 h, and 250 μM 4-OI was pretreated for 12 h before LPS stimulation in experiments.

### Quantification of cytokines and detection of tissue iron

Cytokines, including TNF-α, IL-1β, and IL-6 were quantified according to the kit instruction. The Tissue iron concentrations were also determined as instructed.

### GSH measurement

Total GSH and oxidized glutathione concentration in cells and tissue were determined by a T-GSH/GSSG Detection Assay Kit according to manufacturer protocol.

### Cell viability assay

Cell viability was measured by Trypan Blue staining according to kit instruction.

### Real-time fluorescence quantitative PCR

The total RNA of lung tissues or THP-1 was extracted using TRIpure Total RNA Extraction Reagent (ELK Biotechnology, EP013) and cDNA was synthesized using EntiLink™ 1st Strand cDNA Synthesis Kit (ELK Biotechnology, EP003). Quantitative real-time PCR was performed using EnTurbo™ SYBR Green PCR SuperMix (ELK Biotechnology, EP001). The expression levels of target genes were uniformly normalized to Actin. All primers used in this study were listed in Supplementary Table 1.

### Western blotting

Cells or tissue were lysed in RIPA Lysis Buffer (Servicebio, Wuhan) contained with 1% Phenylmethanesulfonyl fluoride (PMSF, Servicebio). Proteins were separated on 10–15% SDS-polyacrylamide gradient gels and transferred onto PVDF membranes. The 5% skim milk was used to block non-specific binding, and membranes were probed with primary antibodies in 4 °C for 12 h, followed by incubation with anti-rabbit-HRP (1:3000; Proteintech) or anti-mouse-HRP (1:3000; Proteintech) in 37 °C for 2 h. β-actin was selected as the internal reference. The protein bands were visualized with the enhanced chemiluminescence western blotting detection system (Bio-Rad, US).

### ROS measurement

DHE was dissolved in DMSO to a final concentration of 5 mM and further diluted in phosphate-buffered saline (PBS, 1:1000) to a final DMSO concentration of 0.1%, which does not affect ROS generation. THP-1, seeded in a 24-well plate, were incubated by The H2DCFH-DA working solution (10 μM) at 37 °C for 30 min. The frozen sections (10 μm) of lung tissue were incubated with DHE working solution (5 μM) away from light for 30 min at 37 °C. The cell nucleus was stained by Diaminophenyl indole (DAPI). Finally, the fluorescence microscope was used to evaluate the level of ROS.

### Hematoxylin&eosin (H&E) and Masson staining

H&E and Masson staining was performed as our previous study [29]. In brief, multiple fixed-left lungs with formalin were embedded in paraffin and sectioned to 5 μm, followed by staining with hematoxylin-eosin or Masson trichrome stain.

### Lung injury score

The severity of lung injury was estimated using a semiquantitative scoring standard as described previously [30]. Briefly. Four indicators reflecting the severity of lung injury, including alveolar septal thickening, inflammation, hemorrhage, and edema, were blindly evaluated and scored on a scale. The random fields were counted/slide (*n* = 6/group) Alveolar septal thickening, hemorrhage and edema was characterized as follows: absent (score = 0), mild (score = 1), moderate (score = 2), severe (score = 3) and very severe (score = 4); Inflammation was detected by the total number of inflammatory cells/×100 field.

### Immunohistochemical staining

The immunohistochemical staining (IHC) of 4-HNE was performed as previous study [7]. To briefly summarize, the lung paraffin sections were incubated within xylene for dewaxing followed by gradient ethanol solution to hydrate. 3% H_2_O_2_ and 10% goat serum were used to make endogenous peroxidase inactivate and block. Then antibodies against 4-HNE (1:200 dilution with PBS) was used to incubate at 4 °C overnight. At last, anti-rabbit EnVisionTM + /HRP reagent was used to incubated at 37 °C for 1 h. Finally, these sections were observed under a light microscope.

### Immunofluorescence

The immunofluorescence staining of CD68 was performed as previous study [31]. In brief, the lung paraffin sections were dewaxed, hydrated, antigen repaired, and circled. Then sections were incubated with anti-CD68 (1:200) away from light at 4 °C overnight. Next, the goat anti-rabbit secondary antibody (1:200) was used to incubate for 1 h. After DAPI was used to stain the cell nucleus, a confocal laser microscope (LEICA, Germany) was used to observe protein expression.

### Bioinformatic analysis

Raw and processed data from the common database (GSE82043 and GSE145950). Differential expression analysis of RNA-seq data was performed via DESeq2 [32]. Wald test was used to analyze significance testing for group comparisons. Heat maps were generated using the R package.

### Statistical analysis

All analyses were performed in SPSS 23.0 and GraphPad Prism 8 software. Data were expressed as mean ± standard deviation (SD), The multiple group lung injury score was performed by Kruskal–Wallis Test. Unless otherwise specified, the data were representative of at least three independent experiments, the multiple group comparisons were performed by one-way analysis of variance (ANOVA). A confidence interval of 95% was used for all statistical tests, and *P* < *0.05* was regarded to be statistically significant.

## Supplementary information

Supplementary Table1

Supplementary figures

Supplementary figures legends

## Data Availability

All data that support the findings in this study are available from the corresponding author upon reasonable request.
