# Supplementary material for: Itaconate inhibits ferroptosis of macrophage via Nrf2 pathways against sepsis-induced acute lung injury
Source: Cell Death Discov. 2022 Feb 2;8:43. doi: 10.1038/s41420-021-00807-3 (PMC8810876; doi:10.1038/s41420-021-00807-3)
Supplement: Supplementary file 1 — Supplementary Table1 [file 41420_2021_807_MOESM1_ESM.pdf]

| Primer | | 5'-3' |
|---|---|---|
| H-GCLM | sense | CACAGCGAGGAGGAGTTTCC |
| | antisense | CACAGAATCCAGCTGTGCAAC |
| M-GCLM | sense | TTCGCCTCCGATTGAAGATG |
| | antisense | TGGTTACTATTGGGTTTTACCTGTG |
| H-GPX4 | sense | GGCAAGACCGAAGTAAACTACAC |
| | antisense | ACATATCGAATTTGACGTTGTAGC |
| M-GPX4 | sense | GCTGGGAAATGCCATCAAAT |
| | antisense | TCCTTCTCTATCACCTGGGGCT |
| H-Nrf2 | sense | CAGTCAGCGACGGAAAGAGTA |
| | antisense | CTGGGAGTAGTTGGCAGATCC |
| M-Nrf2 | sense | AGTCGCTTGCCCTGGATATC |
| | antisense | GAACAGCGGTAGTATCAGCCA |
| H-HO-1 | sense | GCCAGCAACAAAGTGCAAGA |
| | antisense | TAAGGACCCATCGGAGAAGC |
| H-HO-1 | sense | GCCAGCAACAAAGTGCAAGA |
| | antisense | TAAGGACCCATCGGAGAAGC |
| H-SLC7A11 | sense | TGTGGGGTCCTGTCACTATTTG |
| | antisense | GATATCACAGCAGTAGCTGCAGG |
| H-SLC7A11 | sense | ATCTTCGATACAAACGCCCAG |
| | antisense | GATAATCGTCTGAACCACTTGGG |
| M-SLC7A11 | sense | GAAATATCAGGTCATTGGTGGAGA |
| | antisense | ATGCTCCTGCTTGAGTATGTCG |
| M-IL6 | sense | CCCCAATTTCCAATGCTCTCC |
| | antisense | CGCACTAGGTTTGCCGAGTA |
| M-IL1b | sense | TCAAATCTCGCAGCAGCACATC |
| | antisense | CGTCACACACCAGCAGGTTATC |
| M-TNFα | sense | GTGCCTATGTCTCAGCCTCTTCTC |
| | antisense | GTTTGTGAGTGTGAGGGTCTGG |
| M-actin | sense | GTCCACCGCAAATGCTTCTA |
| | antisense | TGCTGTCACCTTCACCGTTC |
