# Supplementary figures and images for: Itaconate inhibits ferroptosis of macrophage via Nrf2 pathways against sepsis-induced acute lung injury
Source: Cell Death Discov. 2022 Feb 2;8:43. doi: 10.1038/s41420-021-00807-3 (PMC8810876; doi:10.1038/s41420-021-00807-3)

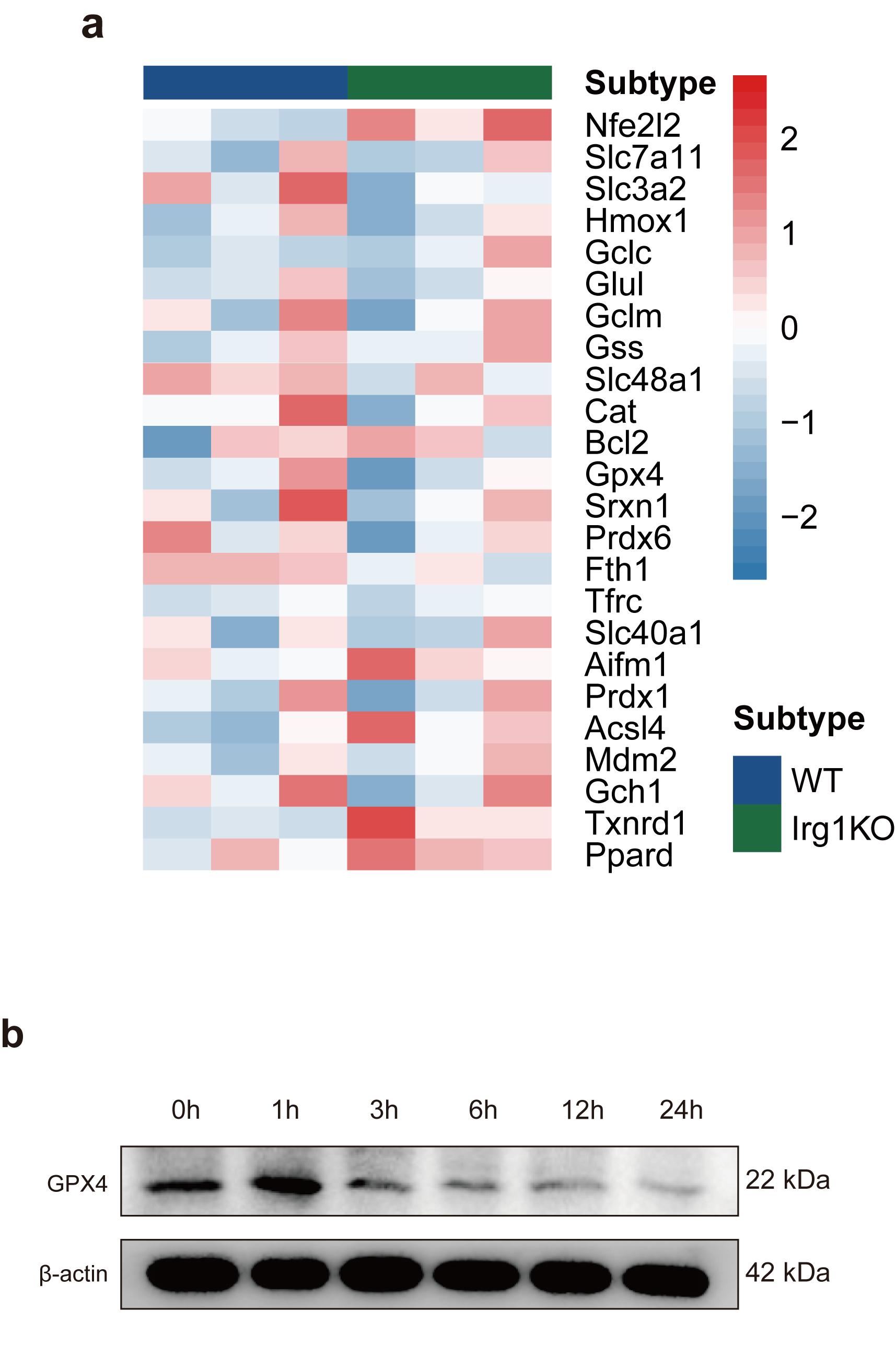

Supplement: Supplementary file 2 — Supplementary figures [file 41420_2021_807_MOESM2_ESM.png]
