# Supplementary material for: Itaconate inhibits ferroptosis of macrophage via Nrf2 pathways against sepsis-induced acute lung injury
Source: Cell Death Discov. 2022 Feb 2;8:43. doi: 10.1038/s41420-021-00807-3 (PMC8810876; doi:10.1038/s41420-021-00807-3)
Supplement: Supplementary file 3 — Supplementary figures legends [file 41420_2021_807_MOESM3_ESM.docx]

**Figure legends**

**Figure S1** a. Genetic difference analysis of ferroptosis associated genes between WT and IRG-KO group. b. Western blots for GPX4 of THP-1 cells at 0h, 1h, 3h, 6h, 12h and 24h after LPS stimulation.
